# Response to letter regarding: [Letter to the editor: impact of parathyroid gland classification on hypoparathyroidism following total thyroidectomy with central neck dissection for differentiated thyroid cancer]

**DOI:** 10.1080/07853890.2025.2536206

**Published:** 2025-07-22

**Authors:** Qixuan Sheng, Wei Li, Ping Zhang, Qiang Wang, Chengxiang Shan

**Affiliations:** Department of Thyroid, Breast and Hernia Surgery of Changzheng Hospital Affiliated to Naval Military Medical University, Shanghai, China

Dear Editor

We appreciate the interest that Hassan et al. have shown in our work [1] and thank them for their insightful comments. We also appreciate their recognition of the clinical significance of our parathyroid gland (PG) classification system.

With respect to their concern that having the same surgical team both performing the operative procedures and conducting the PG classification could introduce observer bias, we acknowledge this as a valid consideration. However, our study was designed prospectively and, given the inherent nature of the surgical procedure, blinding of the surgeons was not feasible. Importantly, surgeons responsible for PG classification were not informed of the patients’ postoperative outcomes. Furthermore, each PG was independently classified by two experienced surgeons, and in the case of disagreement, intraoperative photographic documentation was reviewed and discussed collectively to establish a final consensus. In addition, all outcome measures were objective biochemical parameters, assessed by independent observers who had no role in PG classification or the operative procedures, further minimizing the potential for bias.

We recognize the subjectivity involved in visual identification of PGs and agree that this is a limitation of our approach. Nonetheless, we followed a standardized classification process with independent classification by two experienced surgeons, and our inadvertent PG excision rate of 6.7% (9/135) falls within the range considered clinically acceptable. We also fully concur with the authors’ suggestion that advanced imaging techniques, including near-infrared autofluorescence and indocyanine green angiography, may enhance intraoperative PG identification and preservation [2,3]. However, these techniques require specialized equipment and expertise and are time-consuming, which limits their widespread availability in most hospitals, particularly in developing countries [4]. Our simplified PG classification system was developed as a practical alternative that can be readily adopted in a broad range of surgical settings.

While we agree that both PTH and calcium monitoring are essential for evaluating postoperative parathyroid function, serum calcium is influenced by multiple factors, including diet, sunlight exposure, circadian rhythm, and calcium or vitamin D supplementation. In contrast, PTH serves as a more direct indicator of intrinsic parathyroid activity, which informed our focus on PTH normalization in the present study. Indeed, a small subset of patients developed normo-PTH hypocalcemia during follow-up, potentially related to significant discrepancies between pre- and postoperative PTH levels. We appreciate the emphasis on the need for long-term calcium monitoring. In future research, we intend to extend our follow-up period beyond one year and incorporate serial assessments of serum calcium and clinical symptoms to provide a more comprehensive evaluation of long-term parathyroid function.

Finally, we fully support the need for rigorous methodological standards. Going forward, we will strive to follow established reporting guidelines such as CONSORT and pursue multicenter validation studies with larger cohorts and independent blinded assessors. These steps will help enhance the reproducibility, transparency, and generalizability of our findings.

Once again, we appreciate the constructive feedback and the opportunity to respond. The thoughtful comments by Hassan et al. highlight areas where further refinement is possible and will help us improve the design and reporting of future investigations into strategies for preserving parathyroid function.

Sincerely,

Chengxiang Shan and co-authors


Qixuan Sheng, Wei Li, Ping Zhang, Qiang Wang, and Chengxiang Shan *Department of Thyroid, Breast and Hernia Surgery of Changzheng Hospital Affiliated to Naval Military Medical University, Shanghai, China* chengxiangshan@smmu.edu.cn


## Data Availability

This Letter to the Editor does not involve the generation or analysis of new data. Therefore, data sharing is not applicable.
